# A missed injury leading to delayed diagnosis and postoperative infection of an unstable thoracic spine fracture - case report of a potentially preventable complication

**DOI:** 10.1186/1754-9493-5-25

**Published:** 2011-10-14

**Authors:** Hiroyuki Yoshihara, Todd F VanderHeiden, Philip F Stahel

**Affiliations:** 1 Department of Orthopaedic Surgery, Denver Health Medical Center, University of Colorado School of Medicine, 777 Bannock Street, Denver, CO 80204, USA

## Abstract

**Background:**

Patients suffering from polytrauma often present with altered mental status and have varying levels of examinability. This makes evaluation difficult. Physicians are often required to rely on advanced imaging techniques to make prompt and accurate diagnoses. Occasionally, injury detection on advanced imaging studies can be challenging given the subtle findings associated with certain conditions, such as diffuse idiopathic skeletal hyperostosis (DISH). Delayed or missed diagnoses in the setting of spinal fracture can lead to catastrophic neurological injury.

**Case presentation:**

A man struck by a motor vehicle suffered multiple traumatic injuries including numerous rib fractures, a mechanically unstable pelvic fracture, and also had suspicion for an aortic injury. Unfortunately, the upper thoracic segment (T1-5) was only visualized with axial images based on the electronic data. Several days later, a contrast CT scan obtained to check the status of suspected aortic injury revealed T3-T4 subluxation indicative of an unstable extension-type fracture in the setting of DISH. Due to the missed injury and delay in diagnosis, surgery was not performed until eight days after the injury. At surgery, the patient was found to have left T3-T4 facet joint infection as well as infected hematoma surrounding a left T4 transverse process fracture and a traumatic T4 costo-transverse joint fracture-subluxation. Despite presence of infection, an instrumented posterior spinal fusion from T1-T6 was performed and the patient recovered well after antibiotic treatment.

**Conclusion:**

A T3-T4 unstable DISH extension-type fracture was initially missed in a polytrauma patient due to inadequate imaging acquisition, which caused a delay in treatment and bacterial seeding of fracture hematoma. Complete imaging is especially needed in obtunded patients that cannot be thoroughly examined.

## Introduction

Quality care of trauma patients depends on an efficient and systematic approach for correctly diagnosing clinically important injuries. Previous reports show that some diagnoses can be missed especially in the setting of severe trauma and involving multiply injured patients. Obtundation, altered mental states, and coma can lead to situations where historical data gathering and physical examination findings are difficult to obtain and interpret. There exist several imaging modalities to promptly and accurately identify spine injuries. Numerous studies have been done to see which imaging protocol is best for screening of spinal injuries. Much work has been done with regard to clearance of the cervical spine, culminating in a large multi-centre, prospective validation trial by the NEXUS group [1]. Recently, more studies have been reported with regard to clearance of the thoracic/lumbar (T/L) spine [2-5].

In the present paper, we report the case of a patient with DISH who injured the T3-T4 spinal segment. A delay in diagnosis due to inadequate imaging acquisition and data reformation resulted in prolonged surgical intensive care unit (SICU) stay and associated bacteremia. The blood infection seeded a hematoma around the fractured spinal segment which was discovered at the time of spinal stabilization. Proper, prompt, thorough, and accurate imaging may have avoided this infection and the need for prolonged antibiotic treatment. Furthermore, a significant neurological injury was narrowly avoided. This case demonstrates the importance of imaging acquisition in multiply-injured patients, but also serves to demonstrate the reasonable safety of implanting fracture-fixation devices in the spine in the setting of intra-operative discovery of infection.

## Case report

**Figure 1:**
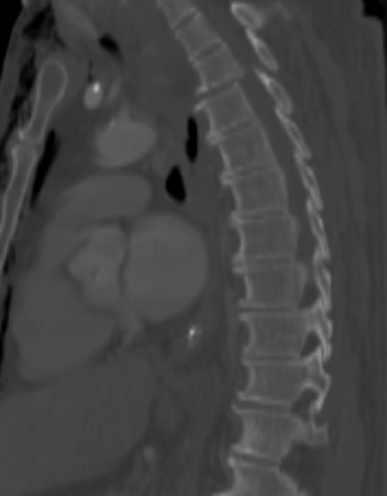
**Sagittal reconstructions of chest CT scan demonstrated a T3-T4 subluxation indicative of a DISH hyper-extension-type fracture**.

**Figure 2:**
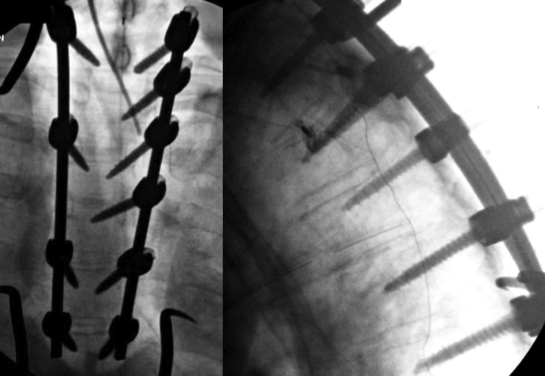
**Posterior spinal fusion from T1 to T6 was performed**. AP and Lateral intra-operative fluoroscopic images show posterior instrumented fusion from T1 to T6.

A 56-year-old, developmentally-delayed, male was struck by a motor vehicle. Emergency medical personnel escorted him to a local hospital. There he was found to have bilateral hemopneumothoraces. Bilateral chest tubes were placed. The medical team also intubated and pharmaceutically paralyzed the patient in preparation for transfer to our level-1 regional trauma center. During transfer, hemodynamic instability required aggressive resuscitation involving blood transfusion. Upon arrival at our institution, it was discovered that the patient had multiple injuries. He suffered a mechanically unstable pelvic fracture, multiple rib fractures, and a wrist injury involving a scaphoid fracture. Additionally, radiographic data from the outside hospital demonstrated concern for an intimal flap tear in the descending aorta. Resuscitation efforts in our SICU established hemodynamic instability. Therefore, his aortic injury was managed non-operatively with observation and cardiovascular monitoring. At that point, there was no concern for spinal injury as the total spinal CT scan did not demonstrate bony injury. Unfortunately, retrospective review would show that the upper thoracic spinal segment was only analyzed with axial images. There were no sagittal or coronal reformations. Nonetheless, the patient underwent uneventful pelvic fracture and wrist fracture fixation. During his SICU course, he developed pulmonary complications including pneumonia. This problem further caused bacteremia evidenced by positive blood cultures. Antibiotic treatment was started. A surveillance CT scan was obtained five days after admission to evaluate for changes to the aortic intimal flap. This scan, utilizing its sagittal and coronal reconstructions, demonstrated a T3-T4 subluxation indicative of a DISH hyper-extension-type fracture (Figure 1). The spine team was, therefore, consulted. The nature of this unstable injury mandated surgical intervention. A posterior spinal instrumentation and fusion approach was selected. At the time of posterior spinal surgical exposure, the patient was found to have left T3-T4 facet joint infection. This was associated with hematoma surrounding a T4 transverse process fracture and a left T4 costo-transverse fracture-subluxation. Intra-operative cultures were obtained. Cultures at the time of surgery grew Haemophilus influenzae (HI) and Methicillin-sensitive Staphylococcus aureus (MSSA). These cultures were consistent with the blood cultures obtained from his episodes of bacteremia related to the lung infection. Intra-operative decision was made to proceed with fixation and fusion secondary to the need for immediate stability of the DISH fracture (Figure 2). Otherwise, the surgery proceeded without complication. Post-operatively, the infectious disease specialist recommended six weeks of culture-directed antibiotics. Three weeks following the surgery, the patient was discharged to a rehabilitation facility. At that stage, he had no wound problems, symptoms or signs of infection, or spinal implant complications. The most recent follow-up imaging demonstrates well fixed spinal implants and no concern for superficial or deep peri-incisional infection. The patient also remains neurologically intact.

## Discussion

Accurate screening of the T/L spine after blunt trauma is important because high-energy collisions result in T/L spine fractures in a significant proportion of patients [6-8]. O'Connor et al. [9] proposes that T/L spine screening should be performed in patients with clinical signs of T/L spine injury, in patients at significant risk of T/L spine injury (high-energy mechanisms) and in patients in whom clinical examination is unreliable (reduced level of consciousness, intoxication, other distracting injuries, c-spine injury, etc.). Early identification of patients with T/L spine injury may limit the potential for developing secondary neurological deficits. Reid et al. [6] reported that patients with T/L spine fractures diagnosed subsequent to admission had a 10.5% incidence of neurological deficit versus a 1.4% incidence if the fractures were diagnosed upon admission. The ideal tool for screening the spine would be accurate and rapid while avoiding added cost or risk to the patient. In terms of assessing injuries of the T/L spine, anteroposterior and lateral radiographs dedicated to the T/L spine is an option. In fact, certain studies state these Xrays are the gold standard [10,11]. Plain film radiographs cost less, expose the patient to lower doses of radiation, and, most importantly, are available in most institutions. However, conventional X-ray evaluation of the spine can result in significant delay in the evaluation of the trauma patient. Berry et al. [2] reported the median time to completion of X-ray studies was 2 hours 48 minutes in their study. Conversely, with chest, abdomen, and pelvis (CAP) CT, the evaluation of the T/L spine is completed shortly after arrival. Berry et al. also studied whether the data obtained from admission CAP CT scans after blunt trauma has utility in T/L spine evaluation. They reported that admission CAP CT obtained as part of the routine trauma evaluation in these high-risk patients is more sensitive than plain radiographs for evaluation of the T/L spine after blunt trauma [2]. The evolution of multidetector computerized tomography (MDCT) technology has revolutionized imaging capabilities alnd allows reconstructing images without additional radiation exposure. Studies have shown that decreased time, cost, and radiation dose are incurred with the implementation of MDCT protocols, eliminating the role of radiographs in screening for T/L spine injuries [12,13].

The use of CT scanning, however, has several disadvantages, including the underdiagnosis of ligamentous injuries and subluxations [14,15]. Kasimatis et al. [16] reported a case of nearly missed cervical dislocation injury where the axial CT images (4-mm slices) were reported as normal. Luckily, lateral cervical X-ray was also obtained and showed the dislocation. In the study of Demetriades et al [17], two patients with subluxations could have been missed with the use of the CT alone. Schenarts et al. [18] also reported three upper cervical spine injuries missed by CT scan but identified by a plain film series. With regard to the T/L spine, Smith et al. [3] studied the reliability of non-reconstructed CT of the abdomen and pelvis as a screening tool for T/L spine injuries. The study occurred in blunt trauma patients with altered mental status. It concluded that reconstruction images do not need to be ordered unless an abnormality is found on the axial CT scan and needs additional elucidation [3]. However, in their study, four fractures were missed in three patients out of a total of 55 fractures. Therefore, they suggested reviewing the scout films or obtaining digital reformatting of sagittal and coronal images to eliminate or reduce the incidence of missed fracture. Brandser et al. [19] suggested reconstructions should be reformatted in the sagittal and/or coronal planes in all situations. These reconstructions are of paramount importance for complete evaluation since fractures may occasionally exist solely in the axial plane. Axial-plane only CT images may, therefore, not demonstrate such fractures [20]. Gestring et al. [10] also suggested a reasonable alternative through using the axial CT scan images supplemented by the scout radiograph to assess patient positioning and alignment in the sagittal plane. As another evaluation modality, MRI is very sensitive and specific, but it can rarely be performed in a patient with multiple injuries during the acute stage of evaluation. This occurs because MRI of the intubated patient may involve a difficult transportation, troublesome monitoring, and an unsafe, time-consuming procedure in this sick population [16].

Our patient's axial spine CT scan was, unfortunately, interpreted as negative for fracture. This certainly appeared to be the situation based on the axial images. Sagittal and coronal reconstruction images had not been reformatted from the original CT data. Therefore, the injury was initially missed. However, retrospective reformatting of images demonstrated the presence of T3-T4 subluxation. Close inspection also showed a transverse process fracture of T4 and T4 costo-transverse fracture-subluxation. Repeat, surveillance CT aortogram, which was accompanied by sagittal and coronal reconstructions, clearly showed the T3-T4 injury, and, thus, a spine consultation was obtained. Another factor may have been that our radiology colleagues, both at the outside hospital and at our institution, were extremely concerned about the potential aortic injury and, therefore, incompletely evaluated the spine. In fact, Bartalena et al. [21] evaluated the prevalence of osteoporotic vertebral fractures in patients undergoing MDCT of the chest and/or abdomen for other reasons, and only 6 out 41 vertebral fractures (14.6%) had been noted in the radiology final report while the remaining 35 (85.45%) had not. Similarly, Muller et al. [22] assessed osteoporotic vertebral deformities in 112 postmenopausal women using axial images and sagittal reformations obtained by MDCT. Osteoporotic vertebral fractures were found in 27 patients but none of these were diagnosed in the official radiology report [22]. This demonstrates that spine surgeon involvement may be beneficial whenever the question of a spinal injury arises. The spine surgeon's familiarity with spinal anatomy, along with knowledge of specific diagnoses that can affect spinal injury and fracture patterns, can help to avoid delayed and/or missed diagnoses.

DISH most commonly affects men who are more than forty years old. Most middle-aged and elderly patients who have DISH are asymptomatic or have mild or moderate restriction of motion [23,24]. DISH is diagnosed, and is distinguished from ankylosing spondylitis, on the basis of several radiographic criteria: flowing calcification and ossification along the anterolateral borders of at least four contiguous vertebral bodies, preservation of the integrity of the intervertebral discs without diminution of disc-space height or other degenerative changes, and the absence of ankylosis in the posterior facets and/or sacro-iliac joints [25]. However, with both these disorders, the spine generally becomes increasingly rigid and osteoporotic, predisposing these patients to fractures that may occur even after a relatively minor traumatic event such as a ground-level fall [26,27]. It is common that the injury extends through both the anterior and posterior columns, giving rise to a grossly unstable spine that places these individuals at significant risk for catastrophic neurologic sequelae [28]. In these patients, the morbidity resulting from spinal fractures approaches 50% and the mortality rate has been reported to be as high as 30% [29,30]. Burkus et al. [31] reported four cases of hyperextension injuries of the thoracic spine in DISH. In all four patients, the osseous elements of the anterior and middle columns remained intact, while fracture of the posterior elements occurred. The patient in this report was similar and had intact osseous elements of the anterior and middle columns, but had a transverse process fracture posteriorly. This scarce osseous involvement may make it difficult to find injuries only with the axial images. In Burkus' report [31], three patients were managed with posterior spinal fusion and all had good outcomes. However, one patient was managed non-operatively with a molded thoracolumbosacral orthosis. Unfortunately, this patient had severe neurological deterioration and non-anatomical alignment of the spine. They advocated such injuries are highly unstable and should be operatively fixated. In our case, the patient was successfully stabilized with posterior spinal fusion, thus avoiding further subluxation and potential neurological sequelae.

At the time of posterior spinal surgical exposure, this patient was found to have left T3-T4 facet joint infection, which was associated with hematoma surrounding a T4 transverse process fracture and a left T4 costo-transverse fracture-subluxation. This suggests bacteremia resulted in hematoma seeding. This may have been prevented with more prompt, accurate diagnosis and earlier treatment. Earlier surgical intervention could have facilitated quicker mobilization while simultaneously allowing improved pulmonary ventilation and avoidance of pneumonia. With regard to treatment of spinal infection, for decades, noninstrumented surgical debridement and in-situ fusion with autograft or allograft has been considered the gold standard for surgical treatment of spinal infection [32-34]. On the other hand, Oga et al. [35] reported that the body's immune system can prevent bacterial attachment to the implant if radical debridement is performed prior to implantation. Rayes et al. [36] found the recurrence rate after instrumentation placed for treatment of spinal infection was 1.74% from the literature review and that of their own cases was 4.2%. This is comparable to the risk of infection after placement of spinal instrumentation in patients with no prior history of spinal infection, which ranges between 2% and 9% [37,38]. Rayes et al. [36] concluded that the use of instrumentation should not be withheld because of risk of introducing foreign material into actively infected sites.

## Conclusion

This patient suffered blunt multi-trauma. Subluxation at T3-T4 was discovered late in his hospital course. Delay in diagnosis occurred because only axial CT images were initially utilized. The injury was found by reformatting CT data to show the sagittal and coronal spinal alignment on a subsequent aortogram. Sagittal and coronal reconstructed CT images may be needed as a routine screening study to assess T/L spine injuries for patients who are obtunded or whose clinical examination is limited. Spine surgeons also need to closely inspect all spine images since their expertise can help avoid missed or delayed diagnoses regardless of the radiology report status. Delayed or missed diagnoses can lead to devastating neurological insults in the setting of spine trauma. This is especially true in stiff spines affected by DISH or AS where injuries usually result in three-column instability. Delayed or missed diagnosis can also result in prolonged hospitalizations and bed-rest which are accompanied by significant potential for complications. Potential problems include bacteremia and sepsis. These mobile bacteria can sometimes settle in less than conspicuous sites and be discovered at inopportune times. Luckily, the necessary addition of foreign material to an unstable, infected spine is usually well tolerated so long as proper debridement and antibiotic therapy are instituted.

## Consent

Written informed consent was obtained from the patient's relatives for publication of this case report.

## Competing interests

PFS has received speaker's honoraria by Synthes (Paoli, PA) and Stryker Spine (Allendale, NJ). The authors declare that they have no competing interests.

## Authors' contributions

HY and TV performed the surgery. HY, TV and PFS wrote the manuscript. All authors contributed to the revisions of the text and approved the final version of this manuscript.
